# Developing and Validating an Instrument to Evaluate Theory-Based Behavioral Antecedents of Consuming a High-Fiber Diet

**DOI:** 10.3390/ijerph17124342

**Published:** 2020-06-17

**Authors:** Paul Branscum

**Affiliations:** Department of Kinesiology and Health, College of Education, Health and Society, Miami University, Oxford, OH 45056, USA; branscpw@miamioh.edu; Tel.: +1-513-529-3022

**Keywords:** obesity, fiber, healthy diet, validity, reliability, reasoned action approach

## Abstract

Obesity is a major public health concern, with low consumption of fiber-rich foods (e.g., fruits and vegetables) commonly cited as a causal factor. The purpose of this study was to evaluate the validity of a survey measuring the constructs of the Reasoned Action Approach under the context of consuming a high-fiber diet. After an initial draft of the survey was developed, it was evaluated by a panel of six experts to establish face and content validity. Next, data were collected from an adult sample (*n* = 878), and psychometric data revealed indices of reliability (Cronbach’s alpha) and validity (confirmatory factor analysis). The average age of adults was 51.5 years (±12.8), and a majority were Caucasian (81%), and women (93%). With regards to construct validity, the model structure had adequate fit (e.g., Comparative Fit Index = 0.960). In addition all items loaded significantly on its corresponding scale. For internal consistency reliability, all Cronbach’s alpha scores were > 0.70. Overall the survey appears to be a promising tool for researchers and practitioners. Understanding the theoretical determinants of fiber consumption will help tie theory together with practice.

## 1. Introduction

**Figure 1:**
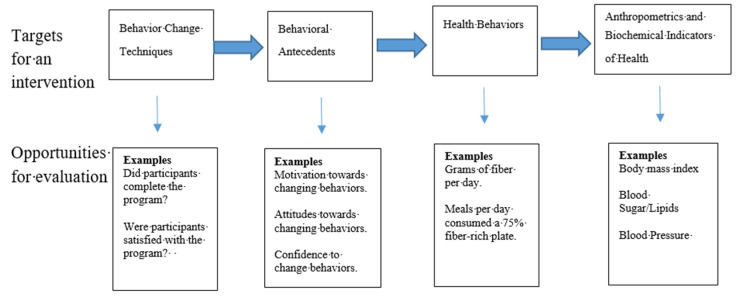
A model of the public health behavior change process and opportunities for evaluation.

Evaluating behavior change programs in public health, health education and health promotion is a critical step in program development and refinement. A proper evaluation can help determine whether program objectives are met, provide accountability to funders, the community, and other stakeholders, and can help contribute to the scientific basis for behavior change interventions. The logic of a behavior change program is depicted in Figure 1, which shows a behavior change program (containing behavior change techniques) is implemented to change behavioral antecedents (e.g., motivation/intentions, attitudes, behavioral confidence, skills, knowledge), which in turn, is done to change health behaviors (e.g., fiber consumption), which ultimately leads to changes in anthropometric and biochemical indicators of health (e.g., BMI, blood pressure, blood sugar).

Obesity is one of the most serious public health concerns. Currently, 39% of men and women are obese worldwide, and in the past 20 years in the United States, obesity increased from 30.5% to 42.4% among adults [1,2]. Alarmingly, the prevalence of severe obesity almost doubled during the same time, from 4.7% to 9.2% [1]. Obesity is associated with a number of metabolic, and mental health risks. Metabolic consequences of obesity include having a higher risk of heart disease, certain types of cancers, stroke, and type 2 diabetes [1]. Obesity among adults has also been associated with having a higher risk of depression and anxiety, and higher discrimination rates in the healthcare setting among numerous health care workers [3,4,5,6].

Consuming a high fiber diet is among the top modifiable risk factors oftentimes promoted to reduce and prevent obesity [7,8]. Fiber has long been promoted for its effect on laxation and having a healthy bowel, but new evidence points to the benefits fiber intake has on preventing and treating chronic diseases, such as type 2 diabetes, cardiovascular disease, and certain types of cancer [9]. Consuming a high fiber diet is also associated with having a lower body weight, and facilitating weight loss [9,10]. For example, in one study with African American women enrolled in a weight loss intervention, while fiber intake was not associated with weight status at baseline, fiber consumption had a strong inverse association with weight status during the 6 and 18 month check in periods of the intervention [11].

In recent years, researchers have evaluated theory-based behavioral determinants of a number of fiber-rich foods, in order to understand what factors contribute to behavior change. For example, in a study using the Theory of Planned Behavior as a framework for predicting fruit and vegetable consumption, researchers found that all of the theory’s constructs (e.g., intentions and attitudes) were significant predictors, accounting for 42% of the variance of the behavior [12]. Additionally, in a separate study, researchers again used the Theory of Planned Behavior as a framework with older adults, and reported which specific beliefs led to the development of attitudes, norms and perceived behavioral control pertaining to fruit and vegetable consumption [13]. Besides fruit and vegetable consumption, whole-grain intake has also been evaluated using similar theory-based approaches [14,15]. However, while individual components of consuming a high fiber diet have been previously evaluated, theory-based determinants of consuming a high-fiber diet as a whole have not been well established. Therefore, the purpose of this study was to develop and validate an instrument to evaluate theory-based behavioral antecedents of consuming a high-fiber diet. In addition, determinants of intentions to consume a high-fiber diet were explored.

## 2. Materials and Methods

### 2.1. Instrument Development

The Reasoned Action Approach (RAA) was utilized as the theoretical basis for the instrument. The RAA has been successfully used for a number of other diet- and health-related behaviors [16,17]. The RAA posits that behaviors are primarily formed by an individual’s intentions/motivation to engage in a behavior, along with an individual’s perceived behavioral control (PBC). Furthermore, the RAA posits that intentions are formed by one’s attitudes about a behavior (including both affective and cognitive attitudes), perceived norms about a behavior (including both descriptive and injunctive norms) and PBC over a behavior (including both autonomy and capacity). The RAA was used as the theoretical model for the survey for a number of reasons. First, the RAA is one of the most highly utilized models in social and behavioral sciences. Second, the RAA is a relatively simple model to understand, and is user-friendly for health practitioners promoting a healthy diet. Finally, the RAA contains a number of different factors health practitioners and researchers can evaluate, thus giving them variety for evaluation plans.

A number of sources were first consulted to assure critical steps of survey development were used to develop the instrument [18,19,20]. First, as suggested by Fishbein and Ajzen, the behavior was defined using the TACT method [target, action, context and time-frame]. Therefore, the instrument was developed for the behavior “Eating a 75% plate at every meal for the next month” [target (a 75% plate), action (eating), context (at every meal) and time-frame (for the next month)]. In the directions of the survey, a ‘75% plate’ was defined as ‘covering 75% of your plate with fiber-rich foods (i.e., fruits, vegetables and beans), and covering the remaining 25% with non-fiber rich foods (i.e., meats, refined grains, desserts)’.

Next, critical constructs of the RAA were identified and the behavior was applied to each construct using examples from a guide published by Fishbein and Ajzen [20]. All items on the instrument were measured on a 7-point semantic differential scale, with two bi-polar sides. Not all of the questions used the same bi-polar sides; therefore, to assure participants understood how to respond to each item, the directions stated “The following questions are measured on scales of 1 to 7. Please answer ONE number on each scale from 1 to 7 that best matches your belief or opinion.” The directions also showed participants how they could interpret the 7-point scale. For example, if the bi-polar words were Strongly Disagree/Strong Agree, the 7-point scale could be interpreted as: Strongly Disagree (1); Somewhat Disagree (2); Slightly Disagree (3); Neither Agree or Disagree (4); Slightly Agree (5); Somewhat Agree (6); Strongly Agree (7). Please see Appendix A for a copy of the survey items and directions. Three items were utilized to evaluate intentions, and were structured as “I <plan/intend/will> to eat a 75% plate at every meal for the next month”. Responses were from Strongly Disagree (1) to Strongly Agree (7). Please see Appendix A for listing of all of the items used to evaluate intentions.

Six items were utilized to evaluate attitudes, of which three items evaluated instrumental (or cognitive) attitudes, and three items evaluated experiential (or affective) attitudes. The phrase “For me, eating a 75% plate at every meal for the next month is…” was used for all items, and the responses for instrumental attitudes included: Not At All Important (1) to Completely Important (7); Completely Worthless (1) to 100% Valuable (7); and Completely Too Time Consuming (1) to Not Time Consuming At All (7). For experiential attitudes, responses to the phrase included: 100% Frustrating (1) to 100% Enjoyable (7); Completely Aggravating (1) to Entirely Satisfying (7), and Completely Unpleasant (1) to Completely Pleasant (7). Please see Appendix A for listing of all of the items used to evaluate attitudes.

Six items were also utilized to evaluate perceived norms, of which three items evaluated the injunctive normative aspect, and three items evaluated the descriptive normative aspect. An example item for injunctive norms was “Most people who are important to me want me to eat a 75% plate at every meal for the next month”. An example item for descriptive norms was “Most people who are important to me will eat a 75% plate at every meal for the next month.” Responses for both items were from Strongly Disagree (1) to Strongly Agree (7). Please see Appendix A for listing of all of the items used to evaluate perceived norms.

Six items were also utilized to evaluate the final construct, PBC. Three items evaluated the capacity aspect (whether one is capable of doing a behavior), and three items evaluated the autonomy aspect of the construct (whether the behavior is within one’s control if they are capable of the behavior). An example item for capacity was “If I wanted to, I could eat a 75% plate at every meal for the next month.” An example item for autonomy was “Eating a 75% plate at every meal for the next month is completely up to me.” Responses for both items were from Strongly Disagree (1) to Strongly Agree (7). Please see Appendix A for listing of all of the items used to evaluate perceived behavioral control.

After the initial items were developed for the RAA constructs, they were sent to a panel of six experts in varying fields (Page Dobbs and Amir Bhochhibhoya were experts on the Reasoned Action Approach; Adam Knowlden and Christine Hackman were experts on developing and validating surveys; and Amar Kanekar and Amanda Wilkerson were experts on nutrition and diet). The main purpose of the review was to evaluate the face and content validity of the survey. These forms of validity are based on subjective evaluations on whether the survey properly evaluates the constructs it purports to evaluate.

### 2.2. Statistical Analysis

For statistical analyses, scales measuring the RAA constructs (e.g., intentions and perceived norms) were transformed to [−3 to +3] [e.g., indicating strong negative perceived norms (−3) to strong positive perceived norms (+3)]. This transformation was done by summating the items on each scale and dividing the sum by the number of items on the scale. For the complete survey, please see Appendix A.

To establish the validity and reliability of each scale, psychometric testing was conducted. Internal consistency reliability was established using Cronbach’s alpha. For each scale, an alpha score ≥ 0.70 was considered acceptable [18]. Construct validity for each scale was established using the maximum likelihood extraction method of confirmatory factor analysis (CFA). For rare cases of missing data, imputations were made. We hypothesized a 4-factor model to be confirmed for the measurement model of the instrument (intentions, attitudes, perceived norms and PBC). All exogenous variables were allowed to covary. Scales were deemed valid if the model fit indices were appropriate (Root Mean Square Error of Approximation (RMSEA ≥ 0.08); Tucker–Lewis Index (TLI ≥ 0.95), and Comparative Fit Index (CFI ≥ 0.95) as well as, if items significantly loaded on the scale, it was theoretically associated with [18]. All analyses were done using SPSS AMOS (version 22) (IBM Analytics; Armonk, NY, USA).

Linear regression analysis was next used to evaluate determinants of intentions for consuming a fiber-rich diet. In the model, intentions were predicted by the RAA constructs (attitudes, perceived norms and PBC). Assumption testing was completed to test for multicollinearity, homoscedasticity, normality (e.g., to assure no issues for central/midpoint scoring occurred), and outliers.

Data were collected from a list serve of individuals in the Full Plate Living community. This community promotes a high-fiber diet, and individuals on the list serve receives periodic emails with fiber-rich recipes and tips on how to increase fiber intake in meals and snacks. The survey was completed entirely online through Qualtrics; however, a paper copy of the survey was also developed (for a copy of the survey please see Appendix A). Before data collection occurred, approval was granted by the sponsoring Institutional Review Board (#03276e). As an incentive, the first 400 participants were given a $20 gift card.

## 3. Results

### 3.1. Participant Overview

Overall, there were 878 people who responded to the email request and completed the survey. To make sure participants were knowledgeable about eating a ‘75% plate’, they were first asked 11 knowledge based questions: six questions based on whether foods were 25% or 75% foods (e.g., Potatoes, Brown Rice, Almonds, Eggs, Grapes, Bread) and five questions were based on whether specific plates were 75%/25% plates. Knowledge scores were generally high. It was decided, however, to only keep participants who scored at least >50% (6 or more out of 11 questions), to assure all participants had a good understanding of the ‘75% plate’. Therefore, 37 participants were removed from the final sample, leaving a sample size of 841 participants.

Participants were between the ages of 18 and 85 years, with the average age of 51.5 years (+/−12.8). The sample was mostly female (*n* = 781; 92.9%), Caucasian [*n* = 683; 81.2%; African American (*n* = 65; 7.7%); Asian (*n* = 21; 2.5%); Native American/American Indian (*n* = 6; 0.7%); Pacific Islander (*n* = 3; 0.4%); other (*n* = 24; 2.9%); missing cases (*n* = 39)], and were mostly educated with a college degree [*n* = 661; 78.6%; less than high school degree (*n* = 1; 0.1%); high school degree or equivalent (e.g., GED) (*n* = 42; 5.0%); some college but no degree (*n* = 135; 16.1%); missing cases (*n* = 2)]. With regards to the RAA constructs, individuals had neutral to weak/positive intentions, neutral to weak/positive attitudes, neutral perceived norms, and strong/positive PBC (Table 1).

### 3.2. Reliability and Validity Evaluation

The Cronbach’s alpha scores revealed that each scale had acceptable internal consistency reliability. A summary of the reliability statistics can be found in Table 2.

With regards to construct validity, upon examining the initial model fit indices, and item-factor loadings in the CFA analysis, it was determined that the scale had adequate validity. All scales yielded significant factor loadings, and good model fit indices (CFI = 0.960, TLI = 0.952, and RMSEA = 0.063). Standardized and unstandardized parameter estimates can be found on Table 3.

### 3.3. Determinants of Intentions to Consumer a Fiber-Rich Diet

A linear regression model was used to explore determinants of intentions for consuming a fiber rich diet. No issues were found for homoscedasticity (examined by constructing residual plots), multicollinearity (examined using variance inflation factor), or outliers (examined using Cook’s distance). Attitudes (*p* < 0.001), perceived norms (*p* < 0.001) and PBC (*p* < 0.001) explained 37.7% of the variance of intentions. According to standardized beta coefficients, perceived norms was the strongest predictor (β = 0.402), followed by PBC (β = 0.233), and attitudes (β = 0.135) (Table 4).

## 4. Discussion

The purpose of this study was to develop a theory-based instrument to evaluate determinants of consuming a high-fiber diet, and evaluate the validity and reliability of the instrument. The approach used for this instrument was one based on a whole-diet approach, rather than evaluating single components of a diet. As previous reviews have noted, while it may seem simple, defining a ‘healthy diet’ can in fact be a difficult task [21]. The United States Dietary Guidelines, the World Health Organization, and the American Academy of Nutrition and Dietetics all have slightly different variations on what is included in a healthy diet, yet there is no universal definition [21]. A common thread, however, through these different definitions of a ‘healthy diet’ is that it contains an adequate amount of micro and macronutrients, contains enough calories to prevent weight loss, yet not an excess of calories that would promote weight gain, and it would balance fiber-rich foods, such as fruits, vegetables, and whole grains, with other types of foods containing high amounts of salt, sugar and fat. The advantage of evaluating one’s diet using a whole-diet approach, as done in the instrument in this study, is that it sets simple guidelines for every meal (e.g., 75% of your plate should have fiber-rich foods), while being flexible for the types of foods that are used to meet this recommendation. Also, counting calories and food groups throughout the day can be a laborious task for consumers, whereas observing what is on a plate is a simpler task.

Overall, the instrument was found to have a high degree of both validity and reliability for all of the constructs of the theory. This type of research is important, as others have noted surveys used in public health, and social and behavioral research, are oftentimes not evaluated to this standard [19,22]. Furthermore, survey development and validation is an essential skill for health educators and public health professionals, as outlined in the core competencies in the field [19]. Yet, despite the need for validity and reliability assessment, a review of the top health education and promotion journals revealed that many times researchers fail to report measures of validity and reliability, and almost no studies evaluated multiple forms of validity as done in this study [23].

The theoretical basis for which this survey was developed should also be noted as a strength to this study, since it is oftentimes the case that a lack of theory is a limitation in survey development [18,19]. As Sharma [24] notes the use of theory in public health and health education is important, as there are many advantages to using theory over having an a-theoretical approach. Such advantages include: theory gives discernible and measurable outcomes for public health interventions, theory guides the selection of behavior change methods and techniques, theory helps identify the optimal timing for interventions, and theory helps researchers and practitioners understand which elements of an intervention are working or are ineffective.

A review of the literature by an expert advisory group found that in the field of health behavior, the Theory of Planned Behavior (TPB—an iteration of the RAA) was the second most utilized model [25]. The RAA represents an advancement in the TPB, since it recognizes differences in cognitive and affective attitudes, and adds a new source of normative pressure, descriptive norms [20]. Both the TPB and RAA have been shown as useful theories in public health. Meta-analyses of TPB [26] and RAA [16] prospective studies showed that behavioral intentions and PBC explain 19.3% to 30.9% of the variance of health behaviors. Additionally, a meta-analysis on TPB-based health behavior change interventions showed on average they contain a weighted effect size (δ^) of 0.50 [17].

The second purpose of this study was to evaluate the prediction model for behavioral intentions, as predicted by the independent variables attitudes, perceived norms, and PBC. Results showed this set of independent variables predicted a substantial amount of the variance of behavioral intentions (37.7%). Furthermore, perceived norms was the strongest predictor (β = 0.402), followed by PBC (β = 0.233), and attitudes (β = 0.135). These results are similar to what has been observed with the aforementioned meta-analyses on the TPB and RAA [16,26], as well as other studies with adults that evaluated determinants of fruit and vegetable consumption [12], sleep behaviors [21], sugar-sweetened beverage consumption [27], and physical activity [28]. More work should be done however, to evaluate additional determinants of intentions to broaden the scope of the RAA. Potential constructs that are not contained within the RAA, such as moral norms, anticipated regret, and social support, should be considered in future research.

To put these results into recommendations for behavior change interventions health practitioners can utilize, it is important to consider the difference between motivational processes (techniques to enhance intentions by changing attitudes and perceived norms) and implementational processes (techniques to enhance PBC by changing skills and the environment) [17]. From the results found in this study, a mix of motivational and implementation processes are likely warranted. Since perceived norms was the strongest predictor, motivational processes should start with reinforcing normative beliefs that consuming a high-fiber diet is something that others in one’s life wants them to perform, and adding additional beliefs that others in one’s life are currently consuming more fiber-rich foods than they may know. PBC can be enhanced by skills-training for identifying fiber-rich foods, and helping individuals set goals around routinely consuming fiber-rich foods. Processes that can help individuals achieve such goals including action planning (e.g., being specific about what foods the individual will consume, and when), and self-monitoring.

This study has a few limitations that should be addressed. First, all of the responses were based on self-reported data, increasing the changes that the data could be biased, as the beliefs reported might not truly represent participants’ actual beliefs. Participants may have also misinterpreted certain questions and mistakenly provided untrue answers. Second, this study used a convenience sample of adults. This sample was mostly female, educated and Caucasian which have all been shown to influence dietary behaviors. For example, in a sample of adults in the United States, it was shown that women and individuals with a higher socioeconomic status had a higher diet quality [29]. Specific gender dietary differences that have been noted included women shop at the grocery store more often, eat less fast food and take-out meals, and consume more whole-grains, fruit and overall fiber compared to men [30,31]. Caucasian individuals also typically have higher diet quality than other racial and ethnic minorities [32,33]. Therefore, caution should be given when attempting to generalize to other groups, and the instrument should be re-tested for validity and reliability when implemented with other groups. Furthermore, this sample was derived from adults on a list serve of individuals in the Full Plate Living community, a community already promoting a high-fiber diet. Efforts should be made to re-validate this survey with adults who are not already predisposed towards consuming a high-fiber diet. Finally, stability (or test–retest reliability) was not established. Future researchers should consider establishing this additional form of reliability.

## 5. Conclusions

In conclusion, while previous studies have explored determinants of individual components of consuming a high-fiber diet (e.g., fruits and vegetables), the purpose of this study was to explore a whole-diet approach. This approach will be advantageous in the community, since the evaluation is broad and can be done using a brief survey. For example, consider a public health program that promotes a healthy diet, and in the evaluation plan practitioners plan to evaluate the behavioral antecedents of all of the foods they are promoting. Rather than having community participants complete multiple surveys (e.g., one on fruits and vegetables, one on whole-grains), this brief survey will capture the beliefs and cognitions of all of these foods together. Concurrently, theory-based research evaluating health behaviors using reliable and valid surveys is significantly needed. Public health professionals need valid and reliable instruments to be assured their evaluations plans are not full of errors. This survey gives researchers and practitioners another tool in the efforts to reduce and prevent overweight and obesity.

## Figures and Tables

**Table 1:** Reasoned Action Approach (RAA) constructs means/standard deviations (all scores are between −3 and +3).

RAA Construct	Mean (SD)
Behavioral Intentions	0.96 (1.7)
Attitudes towards the behavior	0.90 (1.6)
Perceived Norms about the behavior	0.14 (1.3)
Perceived Behavioral Control over the behavior	1.95 (1.1)

**Table 2:** Reliability for RAA constructs.

RAA Construct	Cronbach’s Alpha
Behavioral Intentions	0.95
Attitudes towards the behavior	0.93
Perceived Norms about the behavior	0.87
Perceived Behavioral Control over the behavior	0.90

**Table 3:** Factor Loadings (β) and unstandardized coefficients (B) for Confirmatory Factor Analysis.

Survey Item	Latent Variable	β	B	SE
I plan to eat a 75% plate at every meal for the next month (Int1)	Intentions	0.942	1	
I intend to eat a 75% plate at every meal for the next month (Int2)	Intentions	0.935	1.005	0.020
I will eat a 75% plate at every meal for the next month (Int3)	Intentions	0.907	1.055	0.023
For me, eating a 75% plate at every meal for the next month is… Not At All Important/Completely Important (ATT1)	Attitudes	0.647	1	
Completely Worthless/100% Valuable (ATT2)	Attitudes	0.733	1.377	0.073
Completely Too Time Consuming/Not Time Consuming at All (ATT3)	Attitudes	0.765	1.264	0.065
100% Frustrating/100% Enjoyable (ATT4)	Attitudes	0.912	1.538	0.069
Completely Aggravating/ Completely Satisfying (ATT5)	Attitudes	0.953	1.658	0.072
Completely Unpleasant/Completely Pleasant (ATT6)	Attitudes	0.892	1.567	0.071
Most people who are important to me want me to eat a 75% plate at every meal for the next month (PN1)	Perceived Norms	0.700	1	
People who are significant to me think it is important for me to eat a 75% plate at every meal for the next month (PN2)	Perceived Norms	0.691	0.844	0.031
Most people whom I respect would support me eating a 75% plate at every meal for the next month (PN3)	Perceived Norms	0.534	0.528	0.038
Most people who are important to me will eat a 75% plate at every meal for the next month (PN4)	Perceived Norms	0.838	1.158	0.056
In the past month, most people like me ate a 75% plate at every meal (PN5)	Perceived Norms	0.713	0.901	0.050
How many people similar to yourself will eat a 75% plate at every meal for the next month? (PN6)	Perceived Norms	0.712	0.808	0.044
If I wanted to, I could eat a 75% plate at every meal for the next month (PBC1)	PBC	0.876	1	
I have the ability to eat a 75% plate at every meal for the next month (PBC2)	PBC	0.911	1.036	0.028
To what extent do you see yourself as capable of eating a 75% plate at every meal for the next month? (PBC3)	PBC	0.865	0.988	0.029
I have <No control/Complete control> over whether I eat a 75% plate at every meal for the next month (PBC4)	PBC	0.607	0.627	0.032
Eating a 75% plate at every meal for the next month is completely up to me (PBC5)	PBC	0.566	0.583	0.033
Whether or not I eat a 75% plate at every meal for the next month is entirely my decision (PBC6)	PBC	0.539	0.557	0.033

**Table 4:** Parameter estimates and model prediction for intentions to consume a fiber-rich diet.

3-Component Model	Adjusted R^2^	Standardized Coefficients β	t-Statistic	*p*-Value
Predicting Intentions	0.377 (total)			
Attitudes		0.135	4.580	<0.001
Perceived Norms		0.402	12.979	<0.001
Perceived Behavioral Control		0.233	7.692	<0.001

## Appendix A

Survey Items for Each Scale

**<Items evaluating knowledge: *Individual Foods*>**

In the first few items, we want you to tell us if the following foods belong on the 75% part of the plate OR the 25% part of the plate. If you do not know the answer, please select ‘Unsure’.

	Almonds	75% Food	25% Food	Unsure
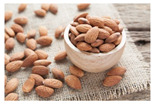
	Sweet Potato	75% Food	25% Food	Unsure
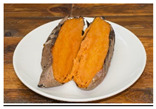
	Brown Rice	75% Food	25% Food	Unsure
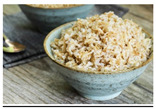
	Grapes	75% Food	25% Food	Unsure
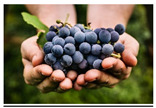
	Scrambled Eggs	75% Food	25% Food	Unsure
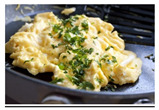
	100% Whole Wheat Bread	75% Food	25% Food	Unsure
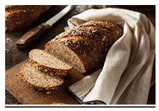

**<Items evaluating knowledge: *Example Plates*>**

Now we would like you to please indicate if the following plates meet the Full Plate Approach (whether it is or is not a 75% plate). If you do not know the answer, please select “Unsure”.

	‘Farmers Breakfast’	This is a 75% plate	This is not a 75% plate	Unsure
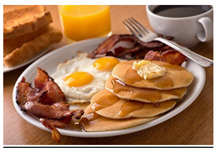
	Spaghetti and Meat sauce	This is a 75% plate	This is not a 75% plate	Unsure
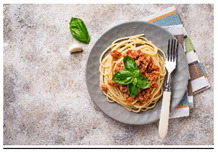
	Fruit and Cereal Bowl	This is a 75% plate	This is not a 75% plate	Unsure
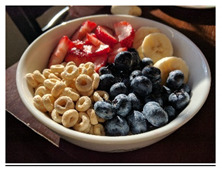
	Beans & Rice w/Salad	This is a 75% plate	This is not a 75% plate	Unsure
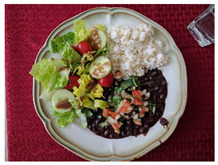
	Baked Salmon w/Tomatoes and Greens	This is a 75% plate	This is not a 75% plate	Unsure
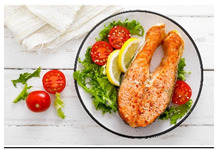

The following questions are measured on scales of 1 to 7. Please answer ONE number on each scale from 1 to 7 that best matches your belief or opinion.

**1**	**2**	**3**	**4**	**5**	**6**	**7**
Extremely	quite	slightly	neither	slightly	quite	extremely
OR						
Strongly	somewhat	slightly	neither	slightly	somewhat	strongly

-------------------------------------------------------------------------------------------------------------

For example, if the question was:

I plan to drink sugary beverages every day in the next week.

Strongly Disagree 1 2 3 4 5 6 7 Strongly Agree

You could think of the responses like…

1 = Strongly Disagree

2 = Somewhat Disagree

3 = Slightly Disagree

4 = Neither Agree or Disagree

5 = Slightly Agree

6 = Somewhat Agree

7 = Strongly Agree

ALSO Remember: “Eating a 75% plate at every meal for the next month” means eating a 75% plate for every meal for the next 30 days, allowing for only 1–2 slip-ups per week.

**<Items evaluating *Intentions*>**

1. I plan to eat a 75% plate at every meal for the next month.
  Strongly Disagree 1 2 3 4 5 6 7 Strongly Agree
2. I intend to eat a 75% plate at every meal for the next month.
  Strongly Disagree 1 2 3 4 5 6 7 Strongly Agree
3. I will eat a 75% plate at every meal for the next month.
  Strongly Disagree 1 2 3 4 5 6 7 Strongly Agree

--------------------------------------------------------------------------------------------------------------

--------------------------------------------------------------------------------------------------------------

**<Items evaluating *Attitudes*>**

4. For me, eating a 75% plate at every meal for the next month is…
  Not At All Important  1 2 3 4 5 6 7 Completely Important
  Completely Worthless 1 2 3 4 5 6 7 100% Valuable
  Completely Too Time  1 2 3 4 5 6 7 Not Time Consuming At All
  Consuming
  100% Frustrating   1 2 3 4 5 6 7 100% Enjoyable
  Completely Aggravating 1 2 3 4 5 6 7 Entirely Satisfying
  Completely Unpleasant   1 2 3 4 5 6 7 Completely Pleasant

--------------------------------------------------------------------------------------------------------------

**<Items evaluating *Perceived Norms*>**

5. Most people who are important to me want me to eat a 75% plate at every meal for the next month.
  Strongly Disagree 1 2 3 4 5 6 7 Strongly Agree
6. People who are significant to me think it is ________ for me to eat a 75% plate at every meal for the next month.
  Not Important At All 1 2 3 4 5 6 7 Completely Important
7. Most people whom I respect would ______ me eating a 75% plate at every meal for the next month.
  Completely Oppose  1 2 3 4 5 6 7 Completely Support
8. Most people who are important to me will eat a 75% plate at every meal for the next month.
  Strongly Disagree 1 2 3 4 5 6 7 Strongly Agree
9. In the past month, most people like me ate a 75% plate at every meal.
  Strongly Disagree 1 2 3 4 5 6 7 Strongly Agree
10. How many people similar to yourself will eat a 75% plate at every meal for the next month?
  None Of Them 1 2 3 4 5 6 7 All of Them

--------------------------------------------------------------------------------------------------------------

**<Items evaluating *Perceived Behavioral* Control>**

11. If I wanted to, I could eat a 75% plate at every meal for the next month.
  Strongly Disagree 1 2 3 4 5 6 7 Strongly Agree
12. I have the ability to eat a 75% plate at every meal for the next month.
  Definitely Not Able 1 2 3 4 5 6 7 Definitely Able
13. To what extent do you see yourself as capable of eating a 75% plate at every meal for the next month?
  Very Incapable 1 2 3 4 5 6 7 Very Capable
14. I have _______ over whether or not I eat a 75% plate at every meal for the next month.
  No Control 1 2 3 4 5 6 7 Complete Control
15. Eating a 75% plate at every meal for the next month is completely up to me.
  Strongly Disagree 1 2 3 4 5 6 7 Strongly Agree
16. Whether or not I eat a 75% plate at every meal for the next month is entirely my decision.
  Definitely False 1 2 3 4 5 6 7 Definitely True

-----------------------------------------------------------------------------------------------------------------
